# The ‘Dark Side’ of Technology in Medical Education

**DOI:** 10.15694/mep.2017.000081

**Published:** 2017-05-02

**Authors:** Laura Delgaty, James Fisher, Richard Thomson

**Affiliations:** 1 Newcastle University; 2 Northumbria Healthcare NHS Foundation Trust

**Keywords:** Technology, Technology Enhanced Learning

## Abstract

This article was migrated. The article was marked as recommended.

Innovation in medical education has almost become synonymous with technology and its use in the field is growing exponentially. The benefits of technology-enhanced learning (TEL) are manifold; however, in this article some of the more contentious, potentially hazardous and oft unexplored aspects of TEL are highlighted - we have termed these the ‘dark side’ of technology in medical education. To further advance the application of technology in medical education, we contend that academics and educators need to turn their attention to the ‘dark side’ to complement the traditional focus on breakthroughs and innovation. Shedding light on the ‘dark side’ of TEL will help educators to develop a more nuanced understanding of the risks and benefits of the technology, that will then facilitate more judicious use of TEL in their teaching. Thus, within this article we outline some key areas for consideration, highlight barriers to exploring these and consider how we might shine a light on the ‘dark side’ of technology in medical education.

## Introduction

Hear the phrase “Innovation in Medical Education” and what comes to mind? Technology is the answer surely, but what was the question? Innovation in medical education has almost become synonymous with technology and the use of technology in the field is growing exponentially. It seems technology is everywhere - articles about it are frequently published in medical education journals, special interest groups are now firmly established, social media use appears ubiquitous amongst learners and technology-related content regularly features in conference proceedings. However, while the technology itself may appear benign, there are drawbacks and dangers associated with some of these innovations; after all, computers don’t plagiarise, impersonate, cyberbully, steal identities or lurk. People do.

We set out to highlight these contentious, potentially hazardous and oft unexplored areas, which we have termed the ‘dark side’ of technology in medical education. We contend that to further advance the application of technology in medical education, academics and educators need to turn their attention to the ‘dark side’ to complement the traditional focus on breakthroughs and innovation. Within this article we outline some key areas for consideration, highlight barriers to exploring these and consider how we might shine a light on the ‘dark side’ of technology in medical education.

## Limitations of TEL research

Although technology is widely used in medical education its effectiveness is still poorly understood ( Wong et al. 2010). Published research is suboptimal and often incomplete, and what has been made public has often failed to inform practice ( Cook 2009). Concerns with published research surrounding technology in medical education include narrow inclusion criteria, incomplete accounting of existing studies, limited assessment of study quality and no quantitative pooling to enable derivation of best estimates of the effect of interventions ( Cook et al. 2011). Although there has been abundant published support of the educational utility of technology, much of what has been made public lacks rigour and robustness. More fundamental still is the issue of what has *not* been made public concerning technology in medical education. As academics and educators with an interest and enthusiasm for technology, we are aware of inconsistencies in the literature, of publication biases towards certain topics, of contentious topics that are not addressed, of negative or null results and of failed educational initiatives that are not disseminated.

## So what exactly is the ‘Dark Side’?

After holding a focus group at Newcastle University, we identified a series of questions, topics and areas. These findings have been categorised and are presented in the table below:

**Table:** 

**The impact of TEL on learners**
***Digital Distraction:***
- Are we breeding shorter attention spans or ‘butterfly minds’?
- Is technology eroding an acceptable knowledge base?
***‘Hacking’ TEL:***
- The ‘gamification’ of simulation by students: how can we ensure the theory to practice gap is bridged?
- Is ‘playing’ the portfolio now a pastime?
**The bad**
Failed medical TEL initiatives: the need for ‘confession corner’?
The perils of learning in an interconnected world: can technology magnify problems as an educator?
Beyond the search engines: the rise of the deep web
**The ugly**
Identity theft in distance learning
Ghost writing: a phantom menace?
Accessibility of illegal/immoral content for academics and students
**The potential for role confusion**
How is the democratisation of knowledge changing the teacher’s role?
Is the assumption that all junior learners are ‘digital natives’ valid?
**The cost of TEL**
How ‘free’ are free apps really?
The cost, burden and sustainability of MOOCs
Are inequalities exacerbated by TEL?
**The ‘who’s watching who’ dilemma**
Lurkers online: Lonely? Or Learning?
The ethics of student and academic surveillance
Security and privacy risks in online learning
Social media safety for staff and students

## Exposing and Exploring the ‘Dark Side’

This ‘dark side’ is presently hidden from academic community’s view - we propose three reasons for this. Firstly, individual educators doing practice-based work tend to perform small-scale local research, which rarely garners the academic esteem needed for publication. Secondly, unflattering aspects and the sensitive nature of these issues, may preclude individuals from acknowledging potential limitations of the ‘cure-all’ that technology in education has become. Thirdly, educators may be loath to share their experiences of unsuccessful technological initiatives, as it is well documented that with failed or delayed implementation of such initiatives, the academic is often blamed for ill will, indolence or ineptitude ( Knight and Trowler 2001). Even if such results are shared, it is recognised that only 10% of published literature suggests null or negative results (Franco et al. 2014). More troublingly, these same authors caution that researchers rarely even write up results that ‘did not work.. and the failure to do so ..adversely affects the universe of knowledge’ (p. 1504). We suggest that by ignoring these topics, we are collectively contributing to the general lack of content knowledge in this field, as the issues remain unobservable to the wider scholarly community.

We contend that sharing these topics would be an invaluable learning experience for medical educators and learners. There is currently no public platform where these issues might be put under the spotlight, in order to inform our practice as educators. We suggest a couple of approaches to address this issue. First, we call on institutions to consider how they can create and develop outlets to improve transparency with null results and with contentious issues in research. Second, having considered these issues within our institution, we have identified priority areas for research, inquiry and case studies, with the goal of exploring and exposing the themes described above. In light of these discussions and reflections, we are hosting a free, inter-professional conference with the intention of exposing, sharing and learning from these less visible issues - you can find out more about it here: http://conferences.ncl.ac.uk/thedarkside/

## Conclusion

We have a responsibility as academics to encourage difficult conversations, to facilitate student and inter-professional discourse and to challenge conventional and accepted views. We call for light to be shed on the ‘dark side’ of technology in medical education. Through the development of a deeper, more balanced understanding of the risks and benefits of the technology, we will be better equipped to shape future implementation of technology-enhanced learning and bring on a new era of enlightenment in medical education.

## Take Home Messages

• The benefits of technology-enhanced learning (TEL) are manifold, but there are contentious, potentially hazardous, oft unexplored aspects of TEL which require greater attention.
• Shedding light on the ‘dark side’ of TEL may help educators to develop a deeper, more balanced understanding of the risks and benefits of the technology.

## Notes On Contributors

Laura Delgaty is a Senior Lecturer at Newcastle University in the Masters of Medical Education Programme with an interest in, and academic responsibilities related to, technology-enhanced learning.

James Fisher is a Consultant Geriatrician at Northumbria Healthcare NHS Foundation Trust, U.K. James is an advocate for the use of technology-enhanced learning in medical education and has developed innovative, simulation-based teaching for geriatric medicine topics. He also co-founded the Association for Elderly Medicine Education.

Richard Kerr Thomson is a Consultant Gastroenterologist at Northumbria Healthcare NHS Foundation Trust, U.K and Clinical Sub-dean at Newcastle University. His interests include learning in the clinical workplace, faculty development, communities of practice and technology-enhanced learning.
