# Supplementary material for: The ‘Dark Side’ of Technology in Medical Education
Source: MedEdPublish (2016). 2017 May 2;6:81. [Version 1] doi: 10.15694/mep.2017.000081 (PMC10885252; doi:10.15694/mep.2017.000081)
Supplement: Peer review history

# Reviewer report: Reviewer response for version 1

**DOI:** 10.21956/mep.19295.r28099

**Published:** 2019-01-09

**Authors:** Mildred López

**Affiliations:** 1 Tecnologico de Monterrey, School of Medicine and Health Sciences

This review has been migrated. The reviewer awarded 3 stars out of 5 I found the article interesting because it addresses very clearly concerns that everyone has. It is written very provocatively and has even already engaged a fair amount of comments. I would emphasize that “the bad” topics that are addressed about problems of quality in research are not specific or exclusive about educational technology, it can be generalized as a medical education problem. Getting your work published it’s already an arduous process, I wonder who would publish a full-paper of a quality study if it provides null or negative results? Maybe we should design specific forums in conferences to provide a dynamic interchange with medical educators, and we could share “what didn’t work”.

Reviewer Expertise:

NA

No decision status is available

# Reviewer report: Reviewer response for version 1

**DOI:** 10.21956/mep.19295.r28093

**Published:** 2018-06-08

**Authors:** Anthony David.M

**Affiliations:** 1 Soho

This review has been migrated. The reviewer awarded 4 stars out of 5 A good read. It is necessary for us to be aware of the dark side of technology too. The various components detailed by the focus groups help us to keep our minds open and be aware of the possibilities.It is a gloomy article but a necessary warning of becoming too technology dependant. Technology should be a tool we can use as we need and not a crutch we lean on.

Reviewer Expertise:

NA

No decision status is available

# Reviewer report: Reviewer response for version 1

**DOI:** 10.21956/mep.19295.r28097

**Published:** 2017-06-23

**Authors:** Laura Delgaty

**Affiliations:** 1 Newcastle University

This review has been migrated. The reviewer awarded 5 stars out of 5 First of all, thank you to all who took the time to respond to our article. This is my first time publishing in this format, so great to see people's thoughts and to respond. Also, so wonderful to have the opportunity to begin a dialogue.Yes, I think that technological initiatives, as expressed by Professor Harden need to be shared so we can learn from them. Personally, I don't see this as gloomy whatsoever! I see it as honest and forward thinking. I mean, those who forget the past are condemned....., right?Secondly, as can be seen by the bios, we are all advocates and promoters of TEL. However, we have seen the effects of blind acceptance and a lack of critical thinking in implementation. I would also suggest to Trudie, that, yes, failed initiatives in TEL for academics can be very different. The roles and responsibilities of academics, historically, have been pretty clear- to write and deliver content (or facilitate learning however one chooses) autonomously. Literature suggests that the advent of TEL has caused role confusion and an erosion of autonomy.Furthermore, before we relax in the comfort of anti plagiarism software keeping our standards safe ( Trudie and Susmita) one can easily create a document that passes the plagiarism check regardless of how much copied material it contains. When there are loopholes that can be exploited, they give the operator a dangerous false assurance that a submission is original.Next, at no point did we suggest, nor do we think this is exclusive to medical education. However, that is the context in which we work and educate, so our focus was directed here. In our upcoming dark side conference, indeed, we have targeted mainstream education at all levels. Finally, I would argue that medical research and medical education traditionally move in different paradigms. Appreciate Ken's point about one potentially informing the other, however, this link, in this context, for us is tenuous. So, overall, the comments are great and have given us (including Jo Matthan from NU who is not listed here) food for thought. This was a brief overview and introduction of our ideas. We will collate the findings after our conference, focus on informing and improving practice, and share this with the medical education, and more broadly education community freely.

Reviewer Expertise:

NA

No decision status is available

# Reviewer report: Reviewer response for version 1

**DOI:** 10.21956/mep.19295.r28094

**Published:** 2017-05-19

**Authors:** Anne McKee

**Affiliations:** 1 -

This review has been migrated. The reviewer awarded 3 stars out of 5 This paper highlights an important issue in both medical education and medical education research. In giving attention to the ‘dark side’ of using technology the authors challenge unexamined enthusiasm for the latest trend or fashion in medical education and highlight the importance of thinking about the drawbacks or limitations of new approaches. In arguing for the reporting of what has not worked, they encourage more transparent and full accounts of what happens within the research process. Medical education has emphasised the importance of doctors learning from mistakes. This papers asks medical education researchers to do the same. For this alone it is worth reading. The paper itself has limitations and many of these have been described. For me, the opportunity to improve the paper is to expand upon the ‘Exposing and Exploring the ‘Dark Side’ which begins to consider the limitations of small scale local research and the state of medical education research as a field of inquiry.

Reviewer Expertise:

NA

No decision status is available

# Reviewer report: Reviewer response for version 1

**DOI:** 10.21956/mep.19295.r28096

**Published:** 2017-05-19

**Authors:** Neel Sharma

**Affiliations:** 1 Albert Einstein College of Medicine, Montefiore Medical Center

This review has been migrated. The reviewer awarded 4 stars out of 5 I congratulate the authors in taking a critical look at TEL. I am surprised we don't see more such pieces when a new teaching or assessment strategy is suddenly brought into the arena. Having initially been drawn to the presumed more wealthy institutes in my nomadic med ed journey I decided to visit those less fortunate and I was amazed by the positive outcomes gained when resources were less plentiful. TEL in many cases is expensive! Money may open doors but does it open our minds?

Reviewer Expertise:

NA

No decision status is available

# Reviewer report: Reviewer response for version 1

**DOI:** 10.21956/mep.19295.r28088

**Published:** 2017-05-16

**Authors:** Susmita Reddy Karri

**Affiliations:** 1 Topiwala national medical college and BYL nair charitable hospital, mumbai. india

This review has been migrated. The reviewer awarded 3 stars out of 5 A good article and it highlights most of the negatives of technology.However, as we all know, that it is always the risk-benefit ratio that we need to consider, and if we look at the scenario in most of the countries, especially the developing/underdeveloped ones, the benefits are far more than the risks. I would have liked if the authors commented on the social status too.Also, I feel that with technology, "ghost writing" or plagiarism is much easy to pick up

Reviewer Expertise:

NA

No decision status is available

# Reviewer report: Reviewer response for version 1

**DOI:** 10.21956/mep.19295.r28087

**Published:** 2017-05-07

**Authors:** Richard Hays

**Affiliations:** 1 James Cook University

This review has been migrated. The reviewer awarded 4 stars out of 5 This is an interesting and timely paper. From the perspective of medical school leadership, it is not uncommon for encounter some students who become involved with not-so-nice posting on social media of poor jokes, ill-advised photos and bad-taste comments. A few spend too much time playing computer games and others store assessment items remembered by colleagues to create 'underground, resources for students. These behaviours can be dangerous for future careers. However, I am not sure that I have seen most of the behaviours or issues listed here. Perhaps my techno-naivety has blinded me! I agree with some of my co-reviewers that the paper is a little gloomy. I agree that big IT systems are expensive, but the potential power of information management seems to make this worthwhile. It would be interesting to read reports from others who have had both negative and positive experiences as we need to develop a balanced view.

Reviewer Expertise:

NA

No decision status is available

# Reviewer report: Reviewer response for version 1

**DOI:** 10.21956/mep.19295.r28092

**Published:** 2017-05-06

**Authors:** Trudie Roberts

**Affiliations:** 1 Leeds Institute of Medical Education

This review has been migrated. The reviewer awarded 3 stars out of 5 An interesting article but far too gloomy. The has always been worry and comment about reduction in attention span and yet then moan that young people spend endless time playing games which require significant time spent on mastering the different levels.. Erosion of an acceptable knowledge base - what is an acceptable knowledge base and where should it reside? Is knowledge to be found only within skull and skin? The whole of knowledge since the dawn of civilisation can now be access on your phone. Assessment of this knowledge and its veracity and validity is one of the many new skills we should be teaching students.Failed TEL initiatives - is this anymore a problem than other failed educational initiatives - i think not. Ghost writing - yes a problem but plagiarism much likely to be picked up now using technology. I could go on.However this piece does set out areas that should form the basis of new research and as such can be applauded particularly if the authors start the ball rolling.....

Reviewer Expertise:

NA

No decision status is available

# Reviewer report: Reviewer response for version 1

**DOI:** 10.21956/mep.19295.r28095

**Published:** 2017-05-05

**Authors:** Ronald M Harden

**Affiliations:** 1 AMEE

This review has been migrated. The reviewer awarded 4 stars out of 5 The use of technology is on today's agenda in medical education and features on lists of trends.. I have recommended this article as it draws our attention to possible problems. I would have liked more details on the issues raised in the table. I like the suggestion that we should publish not only descriptions of successes but also failures. A topic for a future AMEE conference!

Reviewer Expertise:

NA

No decision status is available

# Reviewer report: Reviewer response for version 1

**DOI:** 10.21956/mep.19295.r28091

**Published:** 2017-05-04

**Authors:** Rebecca Grainger

**Affiliations:** 1 University of Otago Wellington

This review has been migrated. The reviewer awarded 3 stars out of 5 This short editorial type piece highlights some considerations for medical educators and their institutions before they dive into TEL. I had hoped that the issues noted in the Table might have been discussed in more detail. I am unsure of the relevance of promoting a conference within this short piece.Some interesting ideas that I would have been keen to have a detailed discussion presented.

Reviewer Expertise:

NA

No decision status is available

# Reviewer report: Reviewer response for version 1

**DOI:** 10.21956/mep.19295.r28089

**Published:** 2017-05-02

**Authors:** Ken Masters

**Affiliations:** 1 Sultan Qaboos University

This review has been migrated. The reviewer awarded 3 stars out of 5 Although the paper is rather pessimistic, it does set out to warn about problems with technology in medical education, and so, is necessarily pessimistic. There are few points, however, that need to be taken into account:Some of the problems referred to in the paper are not restricted to medical education research, and are also currently an inherent part of pure medical research. Two examples: 1. Lack of reporting of negative results. This is standard in most medical research; in fact, a known weakness of EBM is that negative results are seldom published, both for the reasons advanced in this paper, and for the fact that most medical (and scientific) journals are loath to publish negative results. This problem is compounded by commercial sponsorships of EBM research.2. Irreproducibility of results. Again, this is a problem in medical research: one of the greatest concerns in EBM is the number of RCT studies in medicine that cannot be reproduced or, worse, show conflicting results. Often these are also a result of extremely narrow inclusion criteria in trials. This is a problem not only of medical education research, but of research in general, especially when dealing with human subjects.So, while the authors are right in their concerns, medical researchers might be encouraged to examine the plank in their own eye before looking to the speck in that of the comparatively new field of technology in medical education. It would have been useful for the researchers to refer to this, as medical research does form an important context for medical education research.On a positive note, the authors have shown a way for some possible solutions. In addition to these, there is another: allowing and encouraging researchers to publish their raw data so that others may compare results at a deeper level than tables, and meta-analyses can use raw data from several studies. Again, however, this is something from which medical research could also benefit, so, again, we look to medical research to set the pace in this. (As a side note, we should also note that MedEdPublish is happy to publish negative medical education research results, so this journal could a wonderful place for these negative results to be shared)In addition, I think the authors have incorrectly used the term “gamification”. Gamification is not the same as “gaming”, which is the manipulation of a system’s rules and processes for dishonest gain. Gamification is the (generally positive) activity in which principles of games are applied to other situations in order to encourage participation and increase understanding (e.g. the computer game Re-mission, which takes the principles of a “shoot ‘em up” game to teach patients about cancer treatment, or using the design of a monopoly board game to teach patients about the value of nutrition, etc.).Related to this, the authors have rather unfairly lumped the gaming of portfolios to the use of technology in medical education. Portfolios have been around for a long time, and were gamed for a long time before e-portfolios were designed. If there is a problem with e-portfolios, then the technology is unlikely to be the problem – the technology is merely the storage container. Finally, I was also somewhat disappointed that the authors seemed to get started, and then the paper ended rather too soon. I would have liked them to expand a little further on their ideas. Nevertheless, an interesting read and food for thought.

Reviewer Expertise:

NA

No decision status is available

# Reviewer report: Reviewer response for version 1

**DOI:** 10.21956/mep.19295.r28090

**Published:** 2017-05-02

**Authors:** Trevor Gibbs

**Affiliations:** 1 AMEE

This review has been migrated. The reviewer awarded 4 stars out of 5 I enjoyed reading this article which looks at TEL from an opposite angle. It bodes healthy that we look towards the negative effects of new educational approaches and opposed to constantly promoting the benefits.I would agree with one of my co-reviewers that some of these issues relate to non TEL, but that doesn't mean we should avoid the issues in totality. Given that this an opinion piece, I hope that it promotes deeper discussion into the real pros and cons of TEL

Reviewer Expertise:

NA

No decision status is available

# Reviewer report: Reviewer response for version 1

**DOI:** 10.21956/mep.19295.r28098

**Published:** 2017-05-02

**Authors:** Natalie Lafferty

**Affiliations:** 1 University of Dundee

This review has been migrated. The reviewer awarded 4 stars out of 5 Although very short, I think this paper is timely. Whilst it would have been good to see some unpicking of the issues highlighted in your focus group it’s encouraging to see these points reported. There’s a pressing need to see some critical discourse around the use of technology in medical education and higher education more generally. Whilst we can apply and use technology to enhance learning it can also reduce the quality of the learning experience for example death by PowerPoint bullet points or clicky, click, click online learning. It’s fair to say this isn’t always the fault of the technology but rather the individuals or organisations using it. Similarly, the lack of robustness and rigour in the published literature around TEL could be applied to many areas of educational research. There are wider debates going on around the use of technology and the ethics around learner analytics etc. in higher education. I look forward to seeing a growing discourse around #thedarksideofTEL in medical education.

Reviewer Expertise:

NA

No decision status is available
